# Occupational exposure to extremely low-frequency fields and cardiovascular consequences: a systematic review

**DOI:** 10.3389/phrs.2026.1609952

**Published:** 2026-09-25

**Authors:** Elham Raeisi, Sara Maghare Dehkordi, Saeid Yazdanirad, Abdollah Mohammadian-Hafshejani

**Affiliations:** 1 Department of Medical Physics and Radiology Technology, Shahrekord University of Medical Sciences, Shahrekord, Iran; 2 Student Research Committee, Babol University of Medical Sciences, Babol, Iran; 3 Social Determinants of Health Research Center, Shahrekord University of Medical Sciences, Shahrekord, Iran; 4 Department of Epidemiology and Biostatistics, School of Health, Modeling in Health Research Center, Shahrekord University of Medical Sciences, Shahrekord, Iran

**Keywords:** cardiovascular, electromagnetic fields (EMF), environmental exposure, extremely low frequency (ELF), heart

## Abstract

**Objective:**

Exposure to extremely low-frequency (ELF) electromagnetic fields can have harmful effects on human health. The present systematic review study aimed to investigate the relationship between occupational exposure to ELF and cardiovascular outcomes.

**Methods:**

Searches were conducted in six electronic bibliographic databases: Scopus, PubMed, Web of Science, Embase, MEDLINE, and Google Scholar. This search included records up to August 19, 2026. The search strategy used two sets of keywords. Following the selection of proper studies, relevant data were extracted by the reviewers. To assess the risk of bias in the included studies, the Joanna Briggs Institute checklist was used.

**Results:**

Seven of fourteen studies (50 percent) showed that there is an association between ELF field exposure and cardiovascular outcomes. However, the available evidence is insufficient to establish causality. Some of the outcomes reported in the studies included acute myocardial infarction, arrhythmia, hematological parameters, coronary heart disease, and hypertension.

**Conclusion:**

Exposure to ELF fields may be associated with some cardiovascular outcomes. However, the confirmation of this relationship remains ambiguous, and it requires further investigations in future research.

**Systematic Review Registration:**

identifier CRD420251078768.

## Introduction

Extremely low-frequency (ELF) electromagnetic fields are a type of electromagnetic field with frequencies from 1 to 300 Hz [1]. Exposure to these waves is common in occupational environments because it can occur during production, transfer, distribution, and use of electricity [2]. Some effects due to exposure to ELF electromagnetic fields include cerebral cancer, bladder cancer, breast cancer, Alzheimer’s disease, behavioral and mental problems, sleep and wake rhythm disorder, and changes in perception and memory [3]. Compared with these outcomes, the possible cardiovascular effects of ELF exposure have received less attention and remain less clearly understood [4].

Some possible mechanisms have been suggested to explain the association between ELF exposure and cardiovascular diseases. The induced currents due to exposure to these waves can stimulate some cells, such as nerve and muscle cells, and affect electrochemical processes in them [4]. In the extracellular environment, the induced current may be responsible for changes in ion transfer from membranes, voltage-sensitive or other channels, impaired membrane protein function, and hormones or mitogenesis at the cell surface. In addition, these waves can affect intracellular electrical activity [5]. Since normal cardiac function depends on coordinated electrical and physiological processes, changes in these mechanisms could potentially influence cardiovascular function. For example, alterations in cardiac autonomic activity may be reflected in some important indicators of cardiovascular health such as heart rate and variability [5, 6]. Nevertheless, the presence of a possible biological mechanism does not necessarily mean that ELF exposure results in clinically important cardiovascular effects, and the findings from human studies are not yet consistent.

Some studies have examined the relationship between exposure to ELF fields and cardiovascular effects in humans [4, 7, 8]. However, the findings of these studies are contradictory, which may partly be related to variations in exposure intensity, duration of exposure, methods used to assess exposure, characteristics of the study populations, and definitions of cardiovascular outcomes. Such variation makes it difficult to draw a clear conclusion from individual studies.

A few narrative reviews have previously discussed cardiovascular outcomes due to exposure to electromagnetic fields in humans, such as mortality, heart rate, heart rate variability, blood pressure, arrhythmia, and myocardial ischemia [5, 9, 10]. Although these reviews provide useful background information, they were not conducted as systematic reviews. Therefore, they do not provide a comprehensive and reproducible synthesis of the epidemiological evidence. Previous systematic reviews have also either focused on other effects of ELF exposure in humans [11–14] or have been conducted on cardiovascular outcomes *in vivo* experiments [15].

Given the widespread occupational exposure to ELF fields and the uncertainty about their possible cardiovascular effects, a systematic assessment of the available evidence is needed. Therefore, the present systematic review aimed to investigate the relationship between occupational exposure to ELF and cardiovascular outcomes. By bringing together the findings of relevant studies and considering their methodological characteristics, this review aims to provide a clearer picture of the current evidence and identify areas where further research is needed. The findings of this review could provide a clearer picture of the current evidence and inform areas where further research is needed.

## Methods

### Systematic review protocol

This systematic review study has been officially registered with PROSPERO (CRD420251078768) and adheres to the Preferred Reporting Items for Systematic Reviews and Meta-Analyses (PRISMA) guidelines [16]. Additionally, it utilizes the PECO framework—covering Population, Exposure, Comparator, and Outcome—to more effectively illustrate the association between occupational exposure to ELF fields and cardiovascular outcomes [17]. Table 1 provides a summary of the framework details. Figure 1 shows a diagram of PRISMA. The review question was “is occupational exposure to ELF electromagnetic fields associated with an increased risk of cardiovascular outcomes among workers?”.

**TABLE 1:** PECO (Population, Exposure, Comparator, and Outcome) description of the study on extremely low frequency (ELF) and cardiovascular outcomes (Shahrekord, Iran. 2026).

PECO	Evidence
Population	Workers exposed to ELF electromagnetic fields in occupational settings
Exposure	Occupational exposure to ELF electromagnetic fields
Comparator	Workers or participants with a lower level of ELF exposure or no occupational ELF exposure
Outcome	Cardiovascular outcomes due to exposure to ELF fields

**FIGURE 1:**
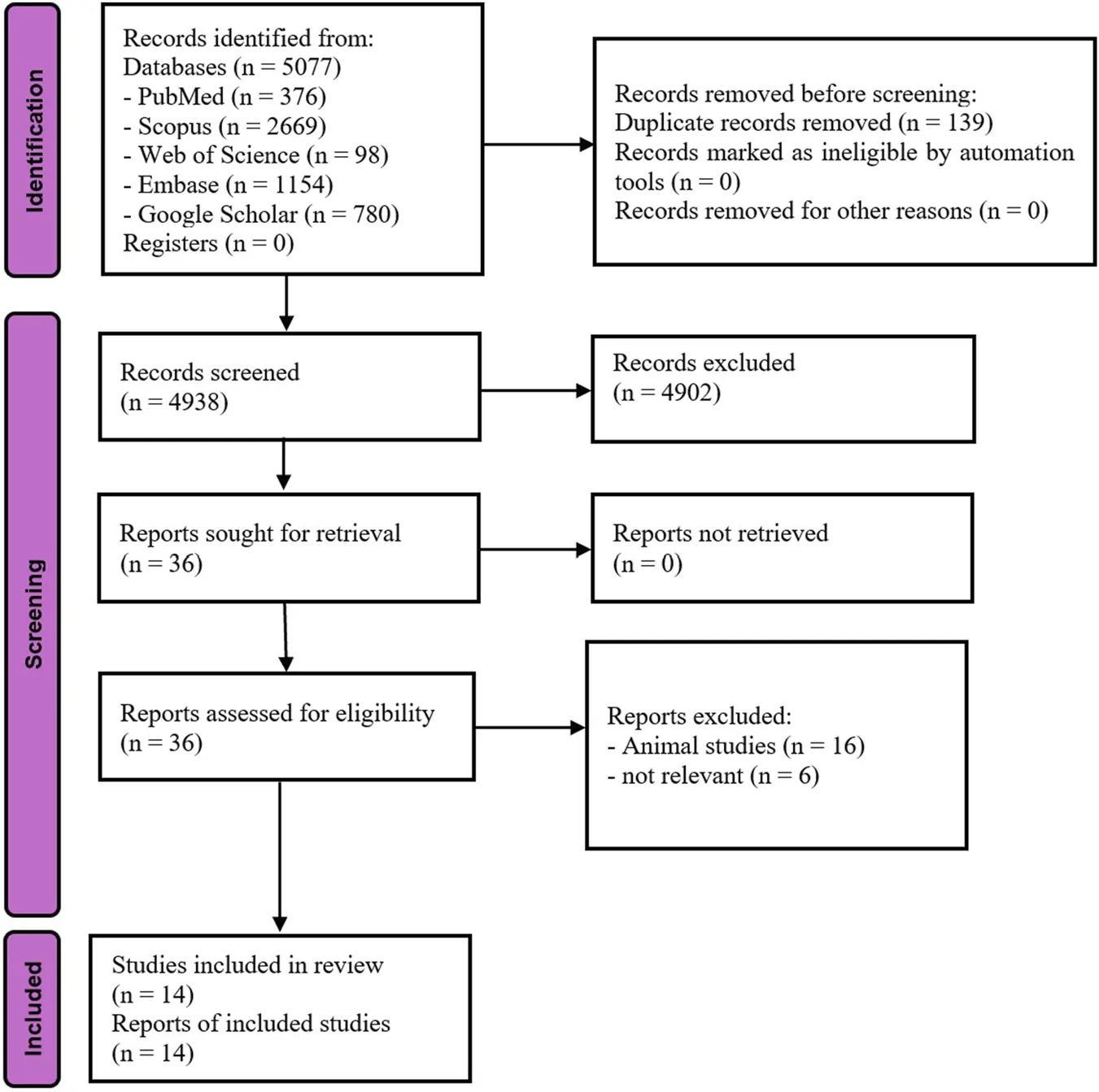
The PRISMA flowchart (Shahrekord, Iran. 2026).

### Search strategy

Two groups of keywords related to ELF field exposure and cardiovascular outcomes were exploited. The first group of keywords consisted of “cardiovascular” or “cardiac” or “vascular” or “heart” or “electrocardiogram” or “HR” or “myocard” or “stroke” or “arterial” or “coronary” or “infarct” or “ischemi” or “blood pressure” or “hypertension”. The second set of keywords was “ELF” or “extremely low frequency” or “extremely low-frequency”. Supplementary Table S1 describes full search strategies used in various databases.

### Data sources

In the current research, a systematic search was conducted in six digital bibliographic databases: Scopus, PubMed, Web of Science, Embase, MEDLINE, and Google Scholar. This search included records up to August 19, 2026.

### Inclusion and exclusion criteria

This systematic review study incorporated all English-language articles that met the following criteria: (1) involved workers exposed to ELF electromagnetic fields in occupational settings; (2) assessed ELF exposure using an objective or clearly described exposure assessment method; and (3) reported at least one cardiovascular outcome, such as cardiovascular disease, cardiovascular mortality, myocardial infarction, ischemic heart disease, arrhythmia, blood pressure, heart rate, or heart rate variability. Different study designs were considered eligible, and no restriction was placed on the year of publication. Studies were excluded if they investigated only non-occupational or environmental exposure to ELF electromagnetic fields or focused on health outcomes unrelated to cardiovascular function, such as sleep problems or psychological outcomes. Review articles, systematic reviews, meta-analyses, conference papers or abstracts without sufficient information, editorials, letters, case reports, and study protocols were also excluded. In addition, studies conducted exclusively in animals, cell cultures, or other non-human models were not considered.

### Study selection

To choose relevant studies, all searched articles were imported to the EndNote software, and duplicate papers were removed. Subsequently, two reviewers (S.Y. and E.R.) independently performed title and abstract screening together. Disagreements between investigators were resolved by consensus using the predefined inclusion and exclusion criteria. Articles considered potentially eligible by either reviewer were retrieved for full-text assessment. Cohen’s kappa coefficient was applied to assess the consistency between screening decisions before consensus.

### Risk of bias assessment

The Joanna Briggs Institute (JBI) evaluation instruments were used to evaluate the risk of bias in the cohort (11 items), experimental (nine items), cross-sectional (eight items), and case-control studies (10 items) [18]. The values of positive responses in the checklist were summed and classified into low bias, moderate bias, and high bias.

### Data extraction

During this phase, two reviewers (S.Y. and E.R.) collected essential data from the selected articles using a standardized data extraction form. Disagreements between reviewers were resolved by consensus. This information included the author’s name, year of publication, country of origin, sample size, study location, type of study, age and gender of the participants, frequency of ELF fields, exposure levels, exposure time, exposure duration, source of ELF fields, tool for ELF field measurement, tool for cardiovascular consequence evaluation, cardiovascular disease type, outcomes, and findings.

### Data analysis

Cohen’s kappa index, utilizing SPSS software, was employed to determine the level of agreement between the raters [19]. During the initial phase of the article selection, the Cohen’s kappa coefficient reached 0.91, and it increased to 0.94 in the subsequent phase that involved the selection of the article information, reflecting a high level of consensus among reviewers.

## Results

### Study selection and characteristics

In this systematic review study, 5077 articles from multiple databases up to August 19, 2026, were identified. Of these, 139 duplicate studies were removed. Following this, two researchers carefully examined the titles and abstracts of the remaining 4938 articles. After a thorough evaluation, 4924 articles were found to be unrelated as they did not meet the required inclusion criteria. Consequently, only 14 articles were included in this study.

Tables 2, 3 display the general and detailed data of the studies included in the systematic review, respectively. Of the 14 studies, five were of a cohort design, four were of a case-control design, four were of an experimental design, and one was of a cross-sectional design. Of the studies, two were from Australia, two from Iran, and one each was from Sweden, the Netherlands, France, Switzerland, China, Japan, South Korea, Germany, Italy, and Romania. These studies were conducted on 49,709 subjects.

**TABLE 2:** The overall information on the articles included in the systematic review (Shahrekord, Iran. 2026).

First author (Year)	Country	Sample size	Location	Study design	Age (years)	Gender	Risk of bias
Håkansson (2003) [7]	Sweden	27,790	Various jobs	Cohort	-	Both	Q1
Koeman (2013) [4]	Netherlands	120,852	Electrical utility workers	Cohort	55–69	Both	Q1
Souques (2021) [20]	France	1479	Electricity transmission network workers	Cohort	62.5	Male	Q3
Röösli (2008) [21]	Switzerland	20,141	Railway workers	Cohort	65	Male	Q1
Liu (2013) [22]	China	374	Workers in automotive industry	Case-control	35	Male	Q2
Fang (2016) [8]	Australia	22	Laboratory	Experimental	20–38	Both	Q1
Kurokawa (2003) [23]	Japan	50	Laboratory	Experimental	20–42	Both	Q1
Weerasinghe (2024) [24]	South Korea	85	Industrial workers	Case-control	20–60	Both	Q2
Moslemi (2023) [25]	Iran	50	Workers in the petrochemical industry	Case-control	35.31	Male	Q1
Griefahn (2002) [26]	Germany	7	Laboratory	Experimental	16–22	Male	Q2
Bagheri hosseinabadi (2019) [27]	Iran	250	Power plant workers	Cross-sectional	20–50	Male	Q1
Cvetkovic (2008) [28]	Australia	5	Laboratory	Experimental	30	Both	Q2
Ptitsyna et al. (1996) [29]	Italy	49,000	Railroad workers and engine drivers	Cohort	20–59	Both	Q2
Danulescu et al. (2012) [30]	Romania	240	High-voltage linesmen and electric train drivers	Case-control	41.49	Both	Q1

Q1: low risk of bias; Q2: moderate risk of bias; Q3: high risk of bias.

**TABLE 3:** The detailed information on the articles included in the systematic review (Shahrekord, Iran. 2026).

First author (year)	Extremely low frequency (ELF) (Hz)	Exposure duration (years)	Exposure time (hours)	ELF sources	Exposure level (μT)	ELF measurement tool	Cardiovascular consequence evaluation tool	Cardiovascular diseases type	Outcome	Findings	Risk of bias
Håkansson (2003) [7]	50–60	6	8	Various occupational sources	<0.10 to >0.3	ELF magnetic field exposure based on the recorded occupation	Causes-of-death database	Acute myocardial infarction, rrhythmia-related death, other ischemic heart disease, and atherosclerosis	Acute myocardial infarction: (relative risk = 1.3, 95% confidence interval: 0.9, 1.9)	Association was not observed between exposure to ELF magnetic fields and deaths related to arrhythmia, ischemic heart disease excluding acute myocardial infarction, or atherosclerosis. However, the authors identified a slight increase in the risk of acute myocardial infarction among individuals in the highest exposure category, along with an exposure-response relationship (p = 0.02)	Q1
Koeman (2013) [4]	50–60	10	8	Various occupational sources	<0.10 to >0.3	Developed ELF-MF job-exposure matrix (JEM)	Database	Total CVD mortality	Hazard ratio: 1.02 (95%CI: 0.99–1.06)	This research found no link between occupational exposure to ELF-MF and deaths due to cardiovascular disease	Q1
Souques (2021) [20]	50–60	22.5	8	Electricity transmission network	0.31 to 1.83	Employment-exposure matrix	Database	Ischemic heart diseases	​	Deaths from ischemic heart disease were less frequent than deaths from other causes	Q3
Röösli (2008) [21]	16.7	31.0	8	Train and railway	5.7–120.5	Commercial gaussmeter	Database	Arrhythmia-related diseases, acute myocardial infarction, atherosclerosis, and sub-acute and chronic coronary heart diseases	Hazard ratio: arrhythmia-related deaths: 1.30 (95%CI: 0.87, 1.93) acute myocardial infarction: 1.56 (95%CI: 1.04, 2.32)	Findings do not support a link between prolonged occupational exposure to intermittent 16.7 Hz magnetic fields and cardiovascular mortality, including deaths due to arrhythmias or acute myocardial infarction	Q1
Liu (2013) [22]	<300	≥2	8	Welding	0.51	EFA-300 field analyzer	Questionnaire	Cardiovascular disease	​	Exposure to extremely low-frequency electromagnetic fields (ELF-EMFs) may influence the nervous, cardiovascular, hepatic, and hematological systems in workers (P < 0.05)	Q2
Fang (2016) [8]	16	-	6	ELF-MF generation system	2.33–6.45	EFA-200 EMF analyzer	Electrocardiogram recording	Electrocardiogram	​	Short-time exposure to ELF-MF can influence electrocardiogram signals (P = 0.003)	Q1
Kurokawa (2003) [23]	50	-	2 min to 12 h	Square coils	20–100	Three-axes flux gate sensor	Electrocardiogram recording	Electrocardiogram	​	Daily exposure to ELF-MF could not have acute effects on the function of the cardiovascular autonomic nervous system (ANS), which regulates heart rate	Q1
Weerasinghe (2024) [24]	50–60	-	24	Industrial equipment	0.19	A portable EMDEX lite device	Automated sphygmomanometer	Blood pressure and heart rate	​	There is a potential association between elevated exposure to ELF-MFs and alterations in systolic blood pressure (p < 0.05)	Q2
Moslemi (2023) [25]	50–60	10.72	8	High voltage power stations	0.08–201.9	Gaussmeter	Medical records	Blood parameters	​	There were no statistically significant differences in the mean values of the variables between the case and control groups (P > 0.05)	Q1
Griefahn (2002) [26]	16.7	-	8	Two helmholtz-coils	0.2	Gaussmeter	ECG recording	Heart rate	​	Exposure to ELF-MF could not be associated with acute effects. However, cumulative effects may occur within the organism, which only appear following repeated exposure	Q2
Bagheri hosseinabadi (2019) [27]	50–60	-	8	Transmission lines, electrical generators, transformers, and distribution grids	29.5	Gaussmeter	Hematological analyzing	Hematological parameters	​	The exposed group showed significantly higher mean values of IL-1b, IL-6, white blood cell count (WBC), red blood cell count (RBC), lymphocyte percentage (lym%), mean corpuscular volume (MCV), platelet count (PLT), and pro-calcitonin (PCT) compared to the unexposed group	Q1
Cvetkovic (2008) [28]	0–100	5 days	5	Programmed control unit, an applicator mattress, and pillow	1–8.3	Gaussmeter	ECG recording	ECG	​	Given the small sample size, these findings do not provide definitive or substantial evidence of potential changes in electrophysiological responses due to ELF exposure	Q2
Ptitsyna et al. (1996) [29]	0.01–10	3	8 h	Electric trains	50 to 280	Computer-based three-component magnetometer	Data recorded in railroad clinics	Cardiovascular diseases (coronary heart diseases and hypertension)	​	The increased risk of coronary heart disease was observed in electric locomotive drivers, which may be linked to their greater occupational exposure to ultra-low frequency magnetic fields	Q2
Danulescu et al. (2012) [30]	50	18.82	8	High-voltage lines and electric train	0.9–21.7	EMF/ELF meter	Detailed clinical, laboratory, and functional tests	Cardiovascular changes (arrhythmias, conduction disturbances, and myocardial ischemia)	Odds ratio: Train drivers: 5.4 (95% CI: 2.18–5.84) high-voltage linesmen 5.18 (95%CI: 1.93–14.24	Occupational exposure to ELF-EMF showed an association with cardiovascular alterations, including arrhythmias, conduction abnormalities, and myocardial ischemia	Q1

ELF-MF: Extremely low-frequency magnetic fields; JEM: Job-exposure matrix; CVD: cardiovascular diseases; CI: confidence interval; IL: interleukin; OR: odds ratio; HR: hazard ratio; Q1: low risk of bias; Q2: moderate risk of bias; Q3: high risk of bias.

Of the studies, four were carried out in laboratories and 10 in real workplaces. These studies were performed on electrical utility workers, electricity transmission network workers, powerplant workers, railway workers, high-voltage linesmen and electric train drivers, workers in the automotive industry, industrial workers, and workers in the petrochemical industry. No study was performed in environmental places.

The age range of the participants was between 20 and 69 years old. Of the studies, six were conducted among males and eight among both males and females.

The ranges of exposure frequency and exposure level were between 0.01 and lower than 300 Hz and between lower than 0.1 and 280 μT. Exposure duration and exposure time of participants were between 5 days and 31 years and between 2 min and 12 h. The sources of ELF exposure also included the electricity transmission network, train and railway, high-voltage lines and electric trains, high-voltage power stations, welding, transmission lines, electrical generators, transformers, distribution grids, industrial equipment, ELF generation system, and other occupational sources.

In the studies, the investigated cardiovascular diseases included acute myocardial infarction, atherosclerosis, arrhythmia-related diseases, sub-acute and chronic coronary heart diseases, ischemic heart diseases, hypertension, and total cardiovascular mortality. Moreover, changes in cardiovascular parameters such as changes in blood pressure, heart rate, electrocardiogram, and other blood and hematological parameters were examined. To determine ELF exposure level, the ELF-MF job-exposure matrix (JEM), gaussmeters, and other direct measurement tools were used. To determine cardiovascular outcomes, various databases, ECG recording, an automated sphygmomanometer, hematological analysis, clinical, laboratory, and functional tests, medical records, and questionnaires were applied.

### Synthesized findings

Based on the findings of the review study, among 14 reviewed articles, seven studies (50.00%) showed that there is a significant association between ELF electromagnetic field exposure and cardiovascular outcomes. Among the seven studies with non-significant findings, three were of a cohort design, three were of an experimental design, and one had a case-control design.

Among studies with low risk of bias, four of eight studies showed that ELF exposure can be associated with cardiovascular outcomes. [7, 8, 27, 30]. Four studies found no significant association [4, 21, 23, 25]. Among studies with a moderate risk of bias, three of five studies indicated that ELF exposure can be associated with cardiovascular outcomes [22, 24, 29]. Four studies showed no significant association [26, 28]. Among studies with a high risk of bias, no study showed the effect of ELF exposure on cardiovascular outcomes [20].

Based on the study design, five cohort studies also examined longer-term occupational exposure and cardiovascular outcomes. Two of these studies found a statistically significant association. Håkansson et al. observed a slight increase in the risk of acute myocardial infarction among workers in the highest exposure category and reported an exposure-response relationship, although associations with arrhythmia-related death, other ischemic heart disease, and atherosclerosis were not observed [7]. Ptitsyna et al. reported an increased risk of coronary heart disease among electric locomotive drivers exposed to ultra-low-frequency magnetic fields [29]. In contrast, Koeman et al. reported no association between occupational ELF magnetic-field exposure and cardiovascular mortality (HR = 1.02, 95% CI: 0.99–1.06) [4]. Similarly, Röösli et al. found no association between prolonged exposure to intermittent 16.7-Hz magnetic fields and cardiovascular mortality among railway workers [21]. Souques et al. also did not find evidence of increased ischemic heart disease mortality among electricity transmission workers [20]. Thus, the cohort evidence was inconsistent, with some evidence suggesting an association with specific cardiovascular outcomes.

Four case-control studies investigated cardiovascular or related physiological outcomes among occupationally exposed workers. Among these studies, three showed a statistically significant association. Danulescu et al. reported associations between ELF exposure and cardiovascular alterations, including arrhythmias, conduction abnormalities, and myocardial ischemia, among train drivers and high-voltage linesmen [30]. Weerasinghe et al. reported an association between ELF exposure and changes in systolic blood pressure among industrial workers [24]. Liu et al. also reported possible cardiovascular effects among workers in the automotive industry [22]. In contrast, Moslemi et al. did not observe statistically significant differences in the assessed cardiovascular-related parameters between exposed and control workers [25]. Overall, the case-control evidence tended to indicate possible associations with cardiovascular function, but the findings were not consistent across studies.

Four experimental studies assessed relatively short-term exposure under controlled conditions. One study indicated a significant association. Fang et al. reported changes in electrocardiographic signals following exposure to pulsed ELF electromagnetic fields [8]. In contrast, Kurokawa et al. found no acute effect of ELF magnetic fields on heart rate or heart rate variability, while Griefahn et al. did not observe acute effects on heart rate [26]. Cvetkovic et al. also reported no definitive changes in electrophysiological responses, although the study had a very small sample size [28]. Therefore, most experimental studies did not demonstrate significant acute cardiovascular effects. These findings should be interpreted separately from those of occupational studies because experimental exposure was generally much shorter and conducted under controlled conditions.

One cross-sectional study investigated workers with long-term occupational ELF exposure. Bagheri Hosseinabadi et al. reported significant differences in several biological parameters between exposed and unexposed powerplant workers [27]. Because the cross-sectional design assesses exposure and outcomes at approximately the same time, however, the temporal relationship between ELF exposure and these changes cannot be established.

### Assessment of risk of bias

In the bias assessment, eight studies had a low risk of bias (57.14%), five had a moderate risk of bias (35.72%), and one had a high risk of bias (7.14%). Table 4 represents the details related to the risk of bias assessment in the reviewed studies.

**TABLE 4:** Details related to the risk of bias assessment in the reviewed studies (Shahrekord, Iran. 2026).

Study	Design	Item 1	Item 2	Item 3	Item 4	Item 5	Item 6	Item 7	Item 8	Item 9	Item 10	Item 11	Risk of bias
Håkansson (2003) [7]	Cohort	Y	Y	Y	Y	Y	Y	Y	Y	Y	Y	Y	Low
Koeman (2013) [4]	Cohort	Y	Y	Y	Y	Y	Y	Y	Y	Y	Y	Y	Low
Souques (2021) [20]	Cohort	Y	N	N	N	N	N	Y	N	N	U	U	High
Röösli (2008) [21]	Cohort	Y	Y	Y	Y	Y	Y	Y	Y	Y	Y	Y	Low
Liu (2013) [22]	Case-control	Y	N	Y	Y	Y	U	N	Y	N	Y	NA	Moderate
Fang (2016) [8]	Experimental	Y	Y	Y	Y	Y	Y	Y	Y	Y	NA	NA	Low
Kurokawa (2003) [23]	Experimental	Y	Y	Y	Y	Y	Y	Y	Y	Y	NA	NA	Low
Weerasinghe (2024) [24]	Case-control	Y	N	Y	Y	Y	N	N	Y	N	Y	NA	Moderate
Moslemi (2023) [25]	Case-control	Y	Y	Y	Y	Y	Y	Y	Y	Y	Y	NA	Low
Griefahn (2002) [26]	Experimental	Y	Y	Y	Y	N	Y	Y	Y	N	NA	NA	Moderate
Bagheri hosseinabadi (2019) [27]	Cross-sectional	Y	Y	Y	Y	Y	Y	Y	Y	NA	NA	NA	Low
Cvetkovic (2008) [28]	Experimental	Y	Y	Y	Y	N	Y	Y	N	N	NA	NA	Moderate
Ptitsyna et al. (1996) [29]	Cohort	Y	N	Y	Y	Y	N	Y	U	N	Y	U	Moderate
Danulescu et al. (2012) [30]	Case-control	Y	Y	Y	Y	Y	Y	Y	Y	Y	Y	NA	Low

Y: yes; N: no; U: unclear; NA: not applicable.

## Discussion

Based on the findings of the present study, among 14 reviewed articles [4, 7, 8, 20–30], seven [7, 8, 22, 24, 27, 29, 30] (50.00%) showed that there is a significant association between ELF electromagnetic field exposure and cardiovascular outcomes. For a better understanding of this issue, it is necessary to first mention the probable effect mechanisms of the ELF field and thereby the characteristics of exposure to these fields. In previous studies, some pathways have been suggested for this relationship.

A possible mechanism is the induction of time-varying magnetic and electric currents based on Faraday’s law V. Electrically excitable cells, such as neurons and muscle fibers, can be directly activated by the induced currents with sufficient intensity [5]. Even at lower intensities, these currents may influence electrochemical activities at the cellular level. Predominantly, the induced currents move in the extracellular space, and they may lead to changes in ion transport across cell membranes, voltage-gated or other types of channels, membrane protein activity, or binding of hormones and mitogens at the cell surface [5]. These findings indicate that ELF fields may be associated with effects at molecular, cellular, or organ levels, potentially leading to biological changes in cardiovascular and neurological tissues. Consequently, exposure to ELF fields can influence both the structure and function of the cardiovascular system, and it may cause cardiovascular diseases [31]. So, exposure to ELF-MF may lead to a change in certain metrics, such as heart rate variability, heart rate, and blood pressure [32]. In a double-blind study, a decrease in heart rate and heart rate variability was observed because of exposure to an ELF field, which may be due to enhanced parasympathetic activity during short-term ELF exposure [33]. Another study on patients with mild to moderate hypertension showed that ELF-EMF exposure can lead to changes in blood pressure [34]. Additionally, clinical evidence has indicated EMF-related vasodilation and improvements in microcirculation [35]. The relationship between changes in heart rate variability, heart rate, blood pressure, and cardiovascular outcomes appears quite strong [36, 37]. Furthermore, exposure to ELF fields has been shown to elevate serum levels of creatine phosphokinase, lactate dehydrogenase, and aspartate aminotransferase enzymes. It also induces oxidative stress and disrupts antioxidant defense mechanisms [38].

The differences found in the study results could be because of differences in factors such as exposure frequency, exposure duration, and exposure level to ELF field. In terms of exposure level, it seems that a higher exposure level can cause more intense consequences. Therefore, the exposure level must be high enough to produce observable effects, while in most studies with non-significant effects, the exposure level was low, between less than 0.1 and 8.3 μT [4, 7, 20, 28]. The currently acceptable limits for human exposure to ELF fields with frequency 50–60 Hz are 1000 μT for occupational exposure and 200 μT for general public exposure [39]. In some reviewed studies, the results also showed a non-significant association despite high levels of exposure [21, 23, 25], which could be due to the effect of other factors such as study design, short exposure duration, features of subjects, and examined consequences [23].

Based on the study design, cohort studies produced mixed results. Case-control studies more frequently reported associations with cardiovascular alterations, whereas most experimental studies did not identify significant acute effects. The single cross-sectional study also reported differences between exposed and unexposed workers. These differences indicate that the evidence should not be interpreted as a single homogeneous body.

The cohort studies, which provide information on longer-term exposure and clinical outcomes, reported mixed findings. Three of the five studies did not find a significant association between ELF exposure and cardiovascular outcomes. These findings suggest that an association between ELF exposure and some specific cardiovascular outcomes is possible, but the evidence from cohort studies is still inconsistent and does not allow a clear conclusion about causality. In most cohort studies [4, 7, 20, 21], despite large sample sizes, databases, a job exposure matrix or table, and modeling were used to determine the exposure level of workers, and personal direct measurement was not performed [20]. This method is expected to primarily result in nondifferential misclassification, which would likely obtain risk estimates nearer to unity, especially among individuals with the highest levels of exposure [7]. Additionally, misclassification due to temporal variations in exposure within occupations may introduce further bias, which most likely attenuates the observed associations [20]. So, the use of these methods instead of direct personal exposure measurement has caused exposure levels to be underestimated. Moreover, the use of data recorded in databases is a limitation of cohort studies [40]. Errors in coding the causes of death are anticipated to be independent of exposure status, which leads to non-differential misclassification of outcomes. This type of misclassification typically results in an underestimation of any true association between exposure and outcome [21].

The case-control studies generally showed somewhat more evidence of a possible cardiovascular consequence. Three of the four studies reported associations between ELF exposure and cardiovascular or related physiological changes, including arrhythmias, conduction abnormalities, myocardial ischemia, and changes in systolic blood pressure. Although these findings suggest that ELF exposure may be related to some aspects of cardiovascular function, the results should be interpreted with caution because case-control studies can be affected by differences between the exposed and comparison groups and by other factors that may not have been fully controlled for.

The experimental studies provide a somewhat different perspective because they examined relatively short-term exposure under controlled conditions. Of the four experimental studies, only one reported a significant cardiovascular effect, namely, a change in electrocardiographic signals. The other studies did not find significant acute changes in heart rate, heart rate variability, or other electrophysiological measures. A non-significant association may be because of short-term exposure, even with high intensity, that cannot sufficiently stimulate physiological responses. Weerasinghe et al. mentioned that prolonged exposure of industrial factory workers to ELF-MFs could be a possible reason for a significant cardiovascular effect [24]. Bagheri Hosseinabadi et al. found that most of the differences in the findings can be attributed to differences in the severity and duration of exposure to ELF-EMFs. However, the levels of ELF field exposure used in most experimental studies are significantly greater than those in occupational settings. Additionally, the exposure duration in these experimental studies generally ranges from hours to months, whereas individuals in occupational environments may experience exposure over several years [27]. Griefahn et al. stated that cumulative effects may occur within the organism after repeated exposure [26]. Therefore, it can be concluded that exposure duration, in addition to the exposure intensity, is an important factor affecting consequences due to exposure to ELF fields. In terms of exposure frequency, the results of the reviewed studies were contradictory. Some studies showed that there is an association between exposure to magnetic fields and heart disease over a wide frequency range of 10–60 Hz [21]. In addition, experimental studies generally focus on short-term physiological responses rather than clinical outcomes such as myocardial infarction or cardiovascular mortality.

Only one cross-sectional study was included in the review. Bagheri Hosseinabadi et al. reported significant differences in several biological parameters between exposed and unexposed powerplant workers. However, because exposure and the outcomes were assessed at the same time, it is not possible to determine whether the exposure occurred before the observed changes. Therefore, the findings provide some indication of a possible association but should not be interpreted as evidence of a causal relationship.

Examined cardiovascular diseases are another important agent affecting the results of the studies. Cardiovascular outcomes refer to the various negative effects that can occur within the cardiovascular system (heart and blood vessels). The results of the reviewed studies showed that significant probable effects due to exposure to ELF fields included changed blood pressure, changed heart rate, acute myocardial infarction, electrocardiogram signals, arrhythmias, hematological parameters, coronary heart disease, and hypertension. No study, however, showed a significant effect of exposure to ELF fields on atherosclerosis and cardiovascular mortality. Therefore, it seems that exposure to ELF fields may be associated with mild to moderate cardiovascular outcomes.

Moreover, personal differences (such as age, gender, and smoking habit), occupational risk factors (such as air pollutants, noise, and high physical activities), and psychosocial factors (such as job and psychological stress) can influence the incidence of cardiovascular diseases. If the role of these confounding factors is not considered in the research, the accuracy of the results obtained will be reduced. In some studies, only the effect of some demographic factors, such as age and gender, as confounders have been eliminated in examining the relationship. This affects the accuracy of the results in the conducted studies.

The identified sources of ELF exposure in the reviewed studies included the electricity transmission network, trains and railways, high-voltage lines and electric trains, high-voltage power stations, welding, transmission lines, electrical generators, transformers, distribution grids, industrial equipment, ELF generation systems, and other occupational sources. Therefore, preventive measures can be focused on these sources. However, these sources are only related to occupational and experimental settings, and there are some sources of exposure to ELF fields in residential environments, such as power lines, electrical wiring, and common household appliances [1]. But no reviewed studies investigated the association between exposure to ELF fields and cardiovascular outcomes in residential settings.

From an occupational health perspective, the possible cardiovascular effects of ELF exposure deserve attention because workers in several occupations may be exposed to these fields for long periods. The studies included in this review reported exposure to these fields in environments such as electricity transmission and distribution, railway systems and electric trains, powerplants, welding, generators, transformers, and other electrical equipment [2]. Although the available studies do not allow us to determine exactly how many workers are exposed to ELF fields at potentially relevant levels, the widespread use of electrical systems means that even a small increase in cardiovascular risk could become important at the population level if the association is confirmed.

Based on the results, the findings are not consistent enough to conclude that ELF exposure is a definite cause of cardiovascular disease or to recommend changes to existing occupational exposure limits. A practical approach is to continue to follow current exposure standards and pay special attention to work environments where exposure is relatively high or occurs over long periods. Regular exposure assessments, identification of major sources, proper maintenance of electrical equipment, use of engineering and administrative measures, and consideration of exposure levels when designing or organizing work environments can help reduce unnecessary exposure. These measures may be particularly beneficial for workers with long-term exposure or who are also exposed to other cardiovascular risk factors [41]. Future research should provide stronger evidence on exposure–response relationships, which could help regulatory and occupational health authorities determine whether existing exposure limits or surveillance policies need to be reconsidered. At present, the findings support greater attention to exposure assessment and preventive workplace practices rather than the introduction of new regulatory limits.

It is also important to consider ELF exposure alongside other occupational factors that can affect cardiovascular health. Occupational noise, air pollution, high physical workload, smoking, and psychosocial stress have much stronger and more proven evidence linking them to cardiovascular disease. Therefore, based on current evidence, ELF exposure should be considered as a possible additional occupational risk factor rather than a proven major cause of cardiovascular disease. Future studies should examine whether the observed cardiovascular effects remain after accounting for these other occupational and personal risk factors.

### Limitations

As a limitation in cohort studies, the use of indirect tools like job-exposure matrices and databases compared to the use of direct valid tools like a gaussmeter, clinical monitoring, and tests can be misleading. Moreover, in experimental studies, the exposure to ELF fields was short-term and so may not have created observable effects in this time period. Furthermore, various factors such as personal, occupational risk, and psychosocial risk factors can influence the incidence of cardiovascular diseases. Therefore, not considering the role of these factors can reduce the accuracy of the results obtained. Moreover, it is required that differences in this association in various populations with different features are reported. Also, the effect of ELF field exposure on healthy people and patients may be different and has not been investigated in the reviewed studies. As another limitation, none of the studies examined the effect of exposure to ELF fields in residential places on cardiovascular diseases, despite there being some sources of this exposure in residential places.

### Conclusions

An association between occupational exposure to ELF electromagnetic fields and cardiovascular outcomes was reported in seven of the 14 studies included in this review. However, the findings were not consistent across the studies, and the current evidence is not sufficient to confirm a causal relationship. The differences in the results of the studies could be because of differences in exposure frequency, exposure duration, and exposure level to the ELF field. Some studies reported associations with acute myocardial infarction, coronary heart disease, arrhythmia, blood pressure, and other cardiovascular-related changes, whereas others found no significant effects. Therefore, although a possible relationship between occupational ELF exposure and cardiovascular health cannot be ruled out, the strength and clinical importance of this association remain uncertain. The findings of this research may be useful to plan strategies to prevent cardiovascular diseases in different settings. However, more studies are needed to investigate the effect of the ELF electromagnetic field with the mentioned characteristics in various environments to confirm these results. Moreover, some confounding factors, such as personal, occupational risk, and psychosocial factors, may influence this association. The roles of these factors must be considered in future studies. Also, differences in these effects between various people with different factors should be examined in future research.

## Data Availability

The original contributions presented in the study are included in the article, further inquiries can be directed to the corresponding author.
