# Supplementary material for: Occupational exposure to extremely low-frequency fields and cardiovascular consequences: a systematic review
Source: Public Health Rev. 2026 Sep 25;47:1609952. doi: 10.3389/phrs.2026.1609952 (PMC13649620; doi:10.3389/phrs.2026.1609952)
Supplement: Supplementary file 1 [file Table1.docx]

**Table S1.** Full search strategies used in various databases

| Database | Search strategy | Number of found papers |
| --- | --- | --- |
| EMBASE | ('cardiovascular disease'/exp OR 'angiocardiopathy' OR 'angiocardiovascular disease' OR 'cardiovascular complication' OR 'cardiovascular disease' OR 'cardiovascular diseases' OR 'cardiovascular disorder' OR 'cardiovascular disturbance' OR 'cardiovascular lesion' OR 'cardiovascular syndrome' OR 'cardiovascular vegetative disorder' OR 'complication, cardiovascular' OR 'disease, cardiovascular' OR 'major adverse cardiovascular event') AND ('low frequency electromagnetic field'/exp OR 'low frequency repetitive transcranial magnetic stimulation'/exp OR 'low frequency ultrasound'/exp OR 'low frequency oscillation'/exp OR 'extremely low frequency magnetic field'/exp OR 'extremely low frequency electromagnetic field'/exp OR 'extremely low frequency pulsed electromagnetic field'/exp OR 'radiofrequency radiation'/exp OR 'radiation, radiofrequency' OR 'radio wave' OR 'radio waves' OR 'radiofrequency radiation' OR 'radiowave') | 1154 |
| PubMed/MEDLINE | (("low frequency electromagnetic field"[All Fields] OR "low frequency repetitive transcranial magnetic stimulation"[All Fields] OR "low frequency ultrasound"[All Fields] OR "low frequency oscillation"[All Fields] OR "extremely low frequency magnetic field"[All Fields] OR "extremely low frequency electromagnetic field"[All Fields] OR "extremely low frequency pulsed electromagnetic field"[All Fields] OR "extremely low-frequency"[All Fields] OR "extremely low-frequency"[All Fields] OR "Radio Wave"[All Fields] OR "Hertzian Waves"[All Fields] OR "Radiowaves"[All Fields] OR "Radiowave"[All Fields] OR "Short Waves"[All Fields] OR "Short Wave"[All Fields] OR "High Frequency Waves"[All Fields] OR "High Frequency Wave"[All Fields] OR "Very High Frequency Waves"[All Fields]) AND ((("Cardiovascular Disease"[All Fields] OR "disease cardiovascular"[All Fields] OR "Cardiac Events"[All Fields] OR "Cardiac Event"[All Fields] OR (("event"[All Fields] OR "event s"[All Fields] OR "events"[All Fields]) AND ("cardia"[MeSH Terms] OR "cardia"[All Fields] OR "cardias"[All Fields]))) AND "c"[All Fields]) OR "Adverse Cardiac Event"[All Fields] OR "Adverse Cardiac Events"[All Fields] OR ("cardiovascular diseases"[MeSH Terms] OR ("cardiovascular"[All Fields] AND "diseases"[All Fields]) OR "cardiovascular diseases"[All Fields] OR ("cardiac"[All Fields] AND "event"[All Fields] AND "adverse"[All Fields])) OR "cardiac events adverse"[All Fields] OR "Major Adverse Cardiac Events"[All Fields])) | 376 |
| ISI | (TS=(“low frequency electromagnetic field” OR “low frequency repetitive transcranial magnetic stimulation” OR “low frequency ultrasound” OR “low frequency oscillation” OR “extremely low frequency magnetic field” OR “extremely low frequency electromagnetic field” OR “extremely low frequency pulsed electromagnetic field” OR “extremely low frequency” OR “extremely low-frequency” OR “Radio Wave” OR “Hertzian Waves” OR “Radiowaves” OR “Radiowave” OR “Short Waves” OR “Short Wave” OR “High Frequency Waves” OR “High Frequency Wave” OR “Very High Frequency Waves”)) AND TS=(“Cardiovascular disease” OR “angiocardiopathy” OR “angiocardiovascular disease” OR “cardiovascular complication” OR “cardiovascular disease” OR “cardiovascular diseases” OR “cardiovascular disorder” OR “cardiovascular disturbance” OR “cardiovascular lesion” OR “cardiovascular syndrome” OR “cardiovascular vegetative disorder” OR “complication cardiovascular” OR “disease cardiovascular” OR “major adverse cardiovascular event”) | 98 |
| SCOPUS | ( ALL ( "Cardiovascular disease" OR "angiocardiopathy" OR "angiocardiovascular disease" OR "cardiovascular complication" OR "cardiovascular disease" OR "cardiovascular diseases" OR "cardiovascular disorder" OR "cardiovascular disturbance" OR "cardiovascular lesion" OR "cardiovascular syndrome" OR "cardiovascular vegetative disorder" OR "complication cardiovascular" OR "disease cardiovascular" OR "major adverse cardiovascular event" ) ) AND ( ALL ( "low frequency electromagnetic field" OR "low frequency repetitive transcranial magnetic stimulation" OR "low frequency ultrasound" OR "low frequency oscillation" OR "extremely low frequency magnetic field" OR "extremely low frequency electromagnetic field" OR "extremely low frequency pulsed electromagnetic field" OR "extremely low frequency" OR "extremely low-frequency" OR "Radio Wave" OR "Hertzian Waves" OR "Radiowaves" OR "Radiowave" OR "Short Waves" OR "Short Wave" OR "High Frequency Waves" OR "High Frequency Wave" OR "Very High Frequency Waves" ) ) | 2669 |
| Google Scholar | (cardiovascular or cardiac or vascular or heart or electrocardiogram or HR or myocard or stroke or arterial or coronary or infract or ischemi or blood pressure or hypertension) and (ELF or extremely low frequency or extremely low-frequency). | 780 |
